# Recurrence of arthroscopic treatment of pigmented villonodular synovitis of the knee: A systematic review and meta‐analysis

**DOI:** 10.1002/jeo2.70169

**Published:** 2025-02-10

**Authors:** Sohrab Keyhani, Mehran Soleymanha, Fardis Vosoughi, Ali Nikibakhsh, Ervin Zadgari, Maryam Mousavi, Robert F. LaPrade

**Affiliations:** ^1^ Bone, Joint, and Related Tissues Research Center Akhtar Orthopedic Training and Research Hospital, Shahid Beheshti University of Medical Sciences Tehran Iran; ^2^ Department of Orthopaedic, Orthopaedic Research Center, Poursina Hospital, School of Medicine Guilan University of Medical Sciences Rasht Iran; ^3^ Department of Orthopedics and Trauma Surgery, Shariati Hospital Tehran University of Medical Sciences Tehran Iran; ^4^ Faculty of Sciences University of Guilan Rasht Iran; ^5^ Department of Complex Knee and Sport Medicine Twin Cities Orthopedics Edina Minnesota USA

**Keywords:** arthroscopic surgery, diffuse pigmented villonodular synovitis (DPVNS), localized pigmented villonodular synovitis (LPVNS), pigmented villonodular synovitis (PVNS), recurrence rate

## Abstract

**Purpose:**

The purpose of this study was to assess the efficacy of arthroscopic intervention on the treatment of pigmented villonodular synovitis (PVNS) patients, with a focus on the potential advantages of this approach in lowering the risk of disease recurrence.

**Methods:**

We performed a systematic review and meta‐analysis following the PRISMA 2020 protocol. Our search encompassed five databases, namely PubMed, Embase, Scopus, Web of Science and Cochrane Library. Statistical analysis was conducted on the extracted data by using the R ver. 4.4.0 software. This study included English‐language observational studies (case series and cohort studies) published up to 31 March 2024, focusing on in vivo human subjects with at least 2 years of follow‐up. Studies with less than 2 years of follow‐up, non‐arthroscopic treatment methods or addressing PVNS in structures other than the knee were excluded.

**Results:**

We identified 24 articles, comprising 7 case series and 17 cohort studies, based on title, abstract, and quality assessments. Approximately 16% (95% confidence interval [CI]: 10.4%–24.75%) of knees that underwent arthroscopic surgery were found to be at risk of recurrence. In line with our expectations, sub‐group analysis comparing recurrence rates among different subtypes of PVNS found that the diffuse subtype exhibited a higher recurrence rate of 19.4% (95% CI: 10.01%–34.15%), compared to the local subtype, which had a recurrence rate of 9.5% (95% CI: 4.47%–19.01%). Based on the meta‐regression analysis, no significant association was found between the recurrence rate and the publication year or patient mean age. However, there was a noticeable rise in the recurrence rate with a longer follow‐up period, indicating a probable correlation between extended follow‐up and increased recurrence rates.

**Conclusion:**

Our findings suggest that arthroscopic surgery for PVNS, particularly for the diffuse subtype, results in a higher recurrence rate compared to the localized subtype. However, the inherent challenges in achieving complete resection through arthroscopy, particularly in cases with extensive disease involvement, may contribute to the observed recurrence rates.

**Level of Evidence:**

Level III systematic review and meta‐analysis.

**Abbreviations**
DPVNS: diffuse pigmented villonodular synovitis
HDI: Human Development Index
LPVNS: localized pigmented villonodular synovitis
PRISMA: Preferred Reporting Items for Systematic Reviews and Meta‐Analysis
PVNS: pigmented villonodular synovitis

## INTRODUCTION

Pigmented villonodular synovitis (PVNS) is a rare benign proliferative disease of synovium which is regarded as a locally aggressive condition affecting joints, bursa membranes and tendon sheaths. The aetiology of the tumour, whether neoplastic or inflammatory, remains unclear. Histologically, PVNS is characterized by hemosiderin deposition, inflammation, multinucleate giant cells and lipid‐laden macrophages [44]. The annual incidence of PVNS is estimated at 1.8 per million, with symptoms classically appearing in the third to fourth decade of life [45]. While PVNS can affect any bodily structure covered by synovium, it most commonly affects the knee (in approximately 70% of cases), followed by the hip and ankle, respectively [25, 38]. Clinically and radiologically, PVNS is classified into two subtypes: the localized subtype (localized pigmented villonodular synovitis [LPVNS]), which presents as a solitary tumour encircled by normal synovium, often in the anterior compartment of the knee, and the diffused type (diffuse pigmented villonodular synovitis [DPVNS]), which involves the entire synovium.

Patients typically exhibit nonspecific symptoms such as joint pain, recurrent non‐traumatic joint effusions, joint locking, reduced range of motion and instability of the affected joint [45, 53]. Magnetic resonance imaging is considered to be the gold standard for evaluating the condition, revealing diffusely thickened synovium with villous finger‐like projections [12, 31]. Considering the rarity of the tumour, coupled with the non‐typical symptoms, patients may receive misdiagnoses of septic arthritis, rheumatic disorders or coagulopathies [53].

The primary treatment for both subtypes of knee PVNS is surgical resection, done either arthroscopically or open [47, 49]. For long‐term treatment, marginal excision of LPVNS is typically recommended, whereas extensive resection is necessary for DPVNS and may still lead to a high likelihood of local recurrence [9]. Alongside surgical resection, the use of intra‐articular radiotherapy or adjuvant external beam has shown promise in effectively controlling the disease locally [14]. Although both techniques have reported satisfactory to excellent functional results, each harbours its limitations. The use of open surgical techniques, which may necessitate both anterior and posterior access, is correlated with more severe wound complications, increased length of hospital stays, protracted rehabilitation, and a heightened risk of post‐operative stiffness [15, 39]. Arthroscopic synovectomy is speculated to expedite healing and minimize complications. However, achieving complete resection through this approach requires a high degree of technical expertise. Notably, arthroscopic techniques are not suitable for the removal of cases involving extra‐articular involvement or large popliteal masses [6, 55]. The combination of arthroscopic and open procedures presents the possibility of achieving complete resection, while minimizing the inherent risks and limitations associated with each approach. However, there is scant objective evidence to substantiate this perspective [28, 53].

Given the rarity of PVNS, conducting randomized controlled trials to assess the impact of various treatment methods is not feasible. Therefore, only observational studies have been viable for evaluation. The purpose of this meta‐analysis was to provide a more comprehensive understanding of the treatment outcomes and recurrence rate of PVNS in the knee. We hypothesized that there would be a reduced recurrence and lower complication rate following the arthroscopic resection.

## MATERIALS AND METHODS

The current study was conducted as a systematic review and meta‐analysis to evaluate the effectiveness of arthroscopic surgery in treating PVNS of the knee by analysing the documented rates of PVNS recurrence following arthroscopic surgery in the existing literature. The findings are presented per the 2020 version of the Preferred Reporting Items for Systematic Reviews and Meta‐Analysis (PRISMA) protocol [36].

### Search strategies

In this review, studies were chosen based on the PICOS principle: Patients = patients who underwent arthroscopic surgery for removal of PVNS, Intervention = Arthroscopic surgery, Control, Outcome = recurrence of PVNS subsequent to the intervention, and Study design = Case‐series and Cohort Studies. Consistent with our study's objectives, the authors surveyed five databases: PubMed, Embase, Scopus, Web of Science and Cochrane Library, and included eligible studies published up to 31 March 2024. The search strategy employed a predefined list of keywords for database. The search strategies are provided in Appendix S1.

The search strategy employed a predefined list of keywords for database search: (((((((“Giant Cell Tumour of Tendon Sheath”[Mesh] OR “Synovitis, Pigmented Villonodular”[Mesh]) AND (knee)) NOT (hip [Title])) NOT (temporomandibular [Title])) NOT (mimic*[Title])) NOT (wrist [Title])) NOT (ankle [Title])) NOT (shoulder [Title]).

Following data collection, duplicate entries were removed using Endnote (v.21). Two researchers then independently screened the articles using the search strategy defined above.

### Inclusion and exclusion criteria

This study included case series and cohort studies published in the English language up to 31 March 2024. Two independent reviewers thoroughly examined the search results and included studies based on predetermined criteria. The utilized criteria included studies conducted on the treatment of PVNS of the knee with a follow‐up period of at least 2 years and human subjects.

Studies with less than 2 years (24 months) of follow‐up period, with treatment methods other than arthroscopic surgery (e.g., adjuvant radiotherapy and open surgery), and addressing other structures affected by PVNS (e.g., ankle and hip) were excluded.

### Quality assessment and data extraction

A quality evaluation of eligible studies was conducted by two researchers using a critical appraisal checklist developed by the Joanna Briggs Institute (2020) https://jbi.global/critical-appraisal-tools.

The checklist comprised 10 points for case‐ series [30] and 11 points for cohort studies and covered various aspects such as the title, abstract, introduction, methods, results, discussion and other pertinent information. Whenever there was any discrepancy, the researchers came to a consensus. Each item was assigned a score accordingly. Furthermore, the first author's name, year of publication, follow‐up period, sample size, recurrence rate, mean age of participants, sex, Human Development Index (HDI) (https://hdr.undp.org/data-center/specific-country-data#) of the countries where the studies originated, and trauma history of the participants were extracted for all eligible studies.

### Statistical analysis

To account for the anticipated between‐studies heterogeneity, we employed a random‐effects model to pool the effect sizes. The restricted maximum likelihood estimator [50] was used to calculate the heterogeneity variance (*τ* ^2^). Furthermore, Knapp‐Hartung adjustments (HK) [22] were applied to determine the 95% confidence interval around the pooled effect. Additionally, Cochrane's *Q* test [7] (with a significance level of less than 0.1) and Higgins & Thompson's *I* ^2^ statistics [17] were used to distinguish studies' sampling error from actual between‐study heterogeneity. Influence analysis was performed to assess the individual effect of each study on the overall results and to find the outliers [51]. Publication bias was assessed visually using a funnel plot and mathematically using Egger's weighted regression test, with a *p* value less than 0.05 indicating statistically significant bias. To further investigate whether the recurrence rate was influenced by time (performed years), a meta‐regression was conducted using the random‐effect model (method of moments). Comprehensive Meta‐Analysis (v.3.7) and R (v.4.4) were used to analyse the data and create visual representations.

## RESULTS

### Study selection

**Figure 1:**
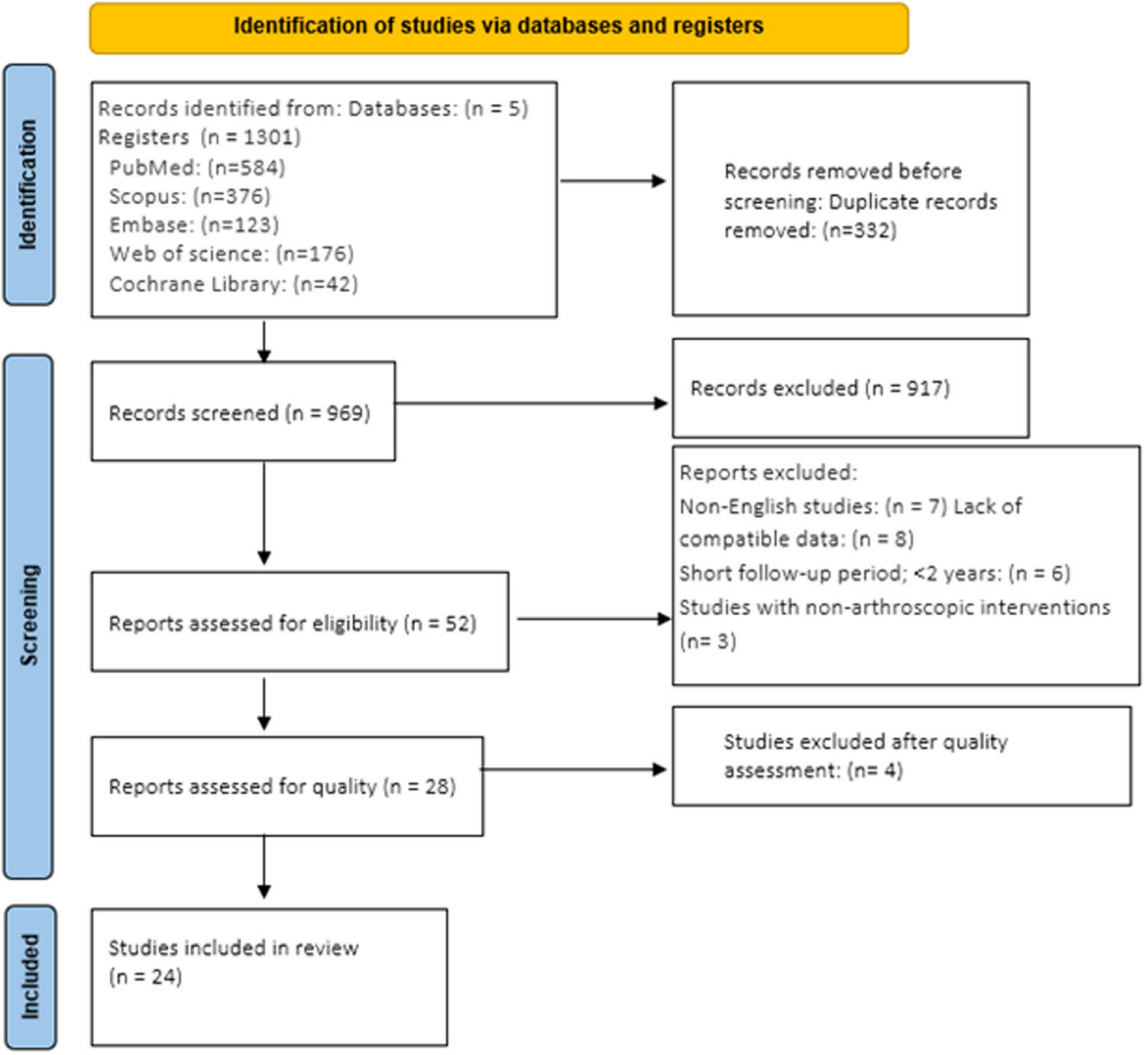
Flowchart of the included eligible studies in the systematic review.^36^

A systematic search across five databases yielded 1301 articles. Following duplicate removal, 969 articles underwent title and abstract screening. Subsequently, 52 articles were selected for full‐text review, resulting in 28 articles for quality assessment. After quality assessment, four studies which were deemed highly biased were removed from the study. Ultimately, 24 articles were included in the final analysis. A detailed diagram of the study collection process is presented in Figure 1.

### Study characteristics

Table 1 presents an overview of the selected studies' general characteristics. The articles were chosen from January 1992 through March 2024. Of the studies analysed, 6 were case series, while 18 were cohort studies. The studies featured varying follow‐up periods, with twelve studies spanning from 5 to 12 years for long‐term monitoring. Medium‐term follow‐ups of 3–5 years were conducted in nine studies, while short‐term studies had less than 3 years of follow‐up. The studies came from various countries, including the United States (6), China (4), Canada (2), Türkiye (2), South Korea (2), Japan (2), India (2), Croatia, Greece, Iran, Italy, the Netherlands, and Taiwan. A summary of the studies is shown in Table 1 [2, 9, 10, 13, 15, 16, 18, 19, 20, 24, 33, 34, 40, 41, 43, 46, 48, 54, 56, 57] and Table 2 [1, 5, 21, 23, 29, 32, 35, 42].

**Table 1:** Characteristics of cohort studies.

No.	Author	Year	Country/HDI	Study population					Recurrence			Follow‐up period (mths)
				Sample size	Localized PVNS	Diffuse PVNS	Mean age (years)	Male/female	Number of recurrences/event rate (%)	Localized recurrence	Diffuse recurrence	
1	Higa et al. [16]	2023	Japan/N/A*	13	7	6	31.8	3/10	0/0%	0	0	35.9
2	Ogilvie‐Harris and Weisleder [34]	1995	Canada/0.876	19	0	19	‐	‐	2/10.53%	‐	‐	38
3	Sharma and Cheng [41]	2009	USA/0.908	18	5	13	35.2	‐	14/77.78%	‐	‐	74.4
4	Xie et al. [54]	2015	China/0.733	118	‐	‐	35.7	‐	26/22.03%	‐	‐	
5	Auregan et al. [2]	2013	France/0.888	23	16	7	41	13/10	2/8.7%	‐	2	84
6	Gu et al. [15]	2014	China/0.732	21	0	21	38	13/8	1/4.76%	‐	1	34.8
7	Jain et al. [18]	2013	India/0.600	40	11	29	44	31/9	12/30%	0	12	84
8	Kerschner et al. [19]	2021	USA/0.921	28	0	28	34.4	10/18	16/57.14%	‐	16	57.66
9	Keyhani et al. [20]	2019	Iran/0.785	21	0	21	32	15/6	2/9.52%	‐	2	60
10	Ogilvie‐Harris et al. [33]	1992	Canada/0.869	25	5	20	38	13/12	6/24%	0	6	55.2
11	Song et al. [43]	2023	China/N/A*	19	0	19	39.8	6/13	2/10.53%	‐	2	36
12	Tie et al. [46]	2023	China/N/A*	18	0	18	‐	8/10	0/0%	‐	0	68
13	van der Heijden et al. [48]	2014	Netherlands/0.931	8	0	16	38.9	5/3	7/87.5%	‐	7	82
14	Yoo et al. [56]	2023	South Korea/N/A*	19	15	14	38.28	14/25	1/5.26%	0	1	56.14
15	Zvijac et al. [57]	1999	USA/0.889	14	2	12	35	7/7	2/14.29%	0	2	42
16	De Ponti et al. [9]	2003	Italy/0.858	19	4	15	59	9/10	0/0%	0	‐	60
17	Dines et al. [10]	2007	USA/0.911	12	12	0	36	‐	0/0%	0	‐	66
18	Georgiannos et al. [13]	2017	Greece/0.880	44	44	0	36	15/11	1/2.27%	1	‐	144
19	Loriaut et al. [24]	2012	France/0.885	20	20	0	47.5	8/12	4/20%	4	‐	75
20	Rhee et al. [40]	2010	USA/0.911	11	11	0	34.1	9/2	2/18.18%	0	‐	112
	Total			510	152	258				5	51	

* As of July of 2024, at the time of writing this article, the latest HDI is of the year 2022.

Abbreviations: HDI, Human Development Index; PVNS, pigmented villonodular synovitis.

**Table 2:** Characteristics of case‐series studies.

No.	Author	Year	Country/HDI	Study population					Recurrence			Follow‐up period (months)
				Sample size	Localized PVNS	Diffuse PVNS	Mean age (years)	Male/female	Number of recurrences/event rate (%)	Localized recurrence	Diffuse recurrence	
1	Cheng et al. [5]	2021	Taiwan/0.723	7	6	1	29.4	1/6	0/0%	0	0	48
2	Nakahara et al. [32]	2012	Japan/0.905	17	‐	17	33.2	10/7	2/11.76%	‐	2	65.4
3	Kubat et al. [23]	2010	Croatia/0.824	13	4	9	28	6/7	1/7.69%	0	1	84
4	Akgun et al. [1]	2003	Türkiye/0.691	7	7	‐	29.14	0/7	0/0%	0	‐	25
5	Kim et al. [21]	2000	South Korea/0.824	11	11	‐	34.6	6/5	0/0%	0	‐	29.9
6	Moskovich and Parisien [29]	1991	USA/0.875	9	9	‐	32.2	3/6	0/0%	0	‐	48
7	Ozalay et al. [35]	2005	Türkiye/0.700	15	15	‐	38.9	3/12	0/0%	0	‐	47.5
8	Shekhar et al. [42]	2017	India/0.640	10	10	‐	33	7/3	0/0%	0	‐	23
	Total			89	62	27		36/53		0	1	

Abbreviations: HDI, Human Development Index; PVNS, pigmented villonodular synovitis.

### Quality appraisal

**Figure 2:**
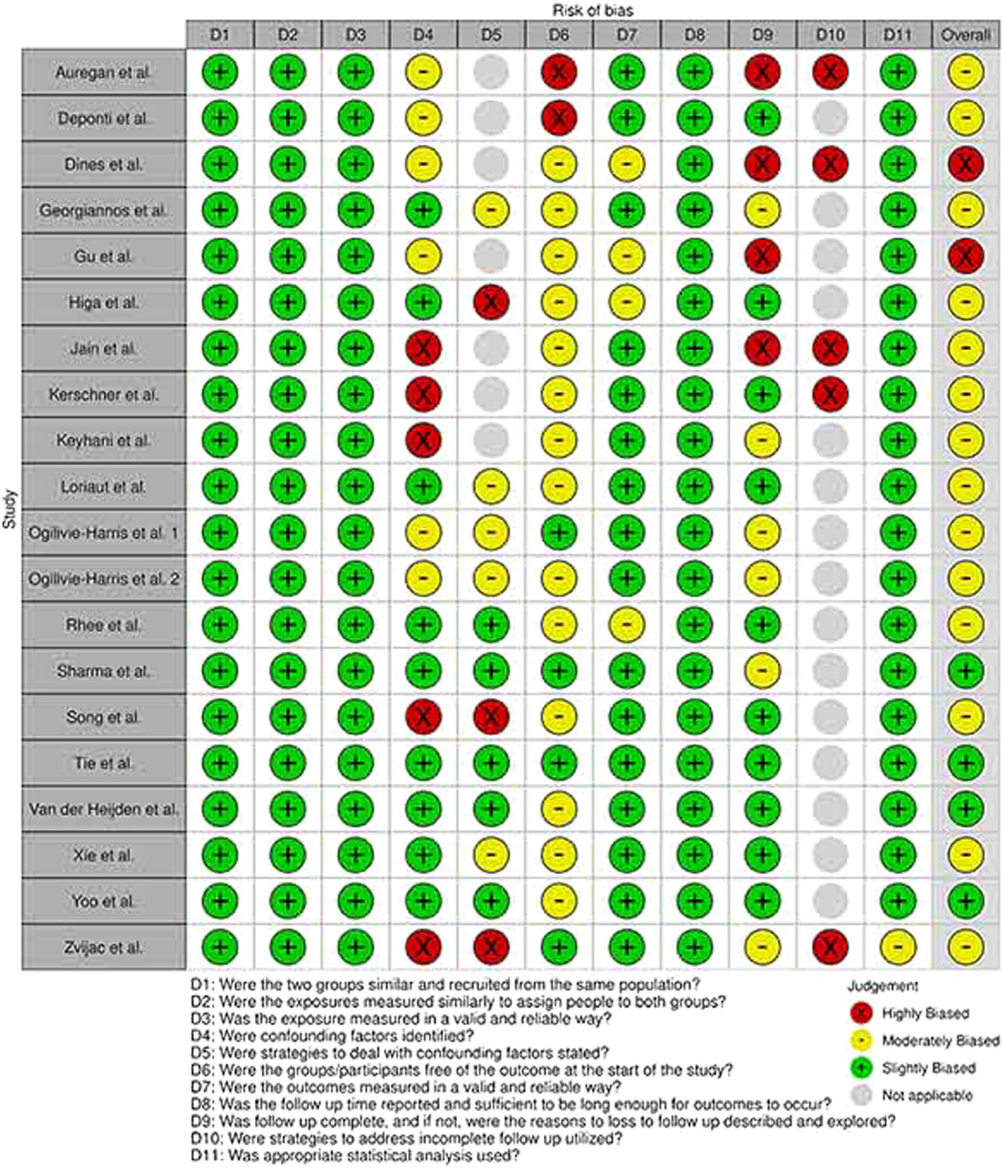
Summary of quality assessment of cohort studies.

**Figure 3:**
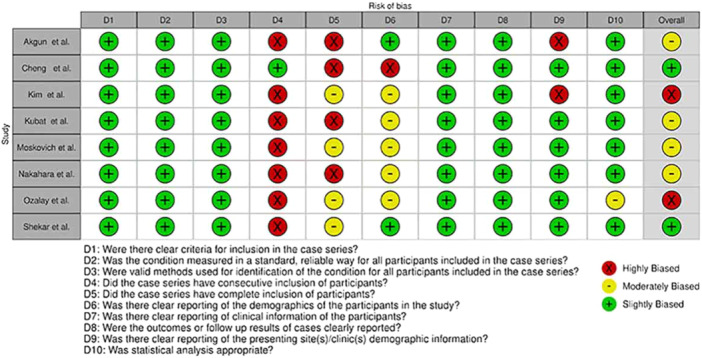
Summary of quality assessment of case‐series studies.

Using relevant checklists, reviewers identified four studies as highly biased (low‐quality)—namely, Kim et al. [21], Ozalay et al. [35], Dines et al. [10] and Gu et al. [15]—and 20 articles as slightly and moderately biased. The visualization of the risk of bias was performed with ROBVIS tool [26]. A summary of quality assessment is presented in Figures 2 and 3.

### Heterogeneity

The between‐study heterogeneity variance was estimated at *τ* ^2^ = 1.2789 (95% confidence interval [CI]: 0.4849–3.2401), with an *I* ^2^ value of 70.2% (95% CI: 54.8%–80.3%). The high value of *I* ^2^ elucidates a substantial between‐study heterogeneity. The results of Cochrane's *Q* test bolster the findings (*Q* = 77.06, df = 23, *p* < 0.0001).

**Figure 4:**
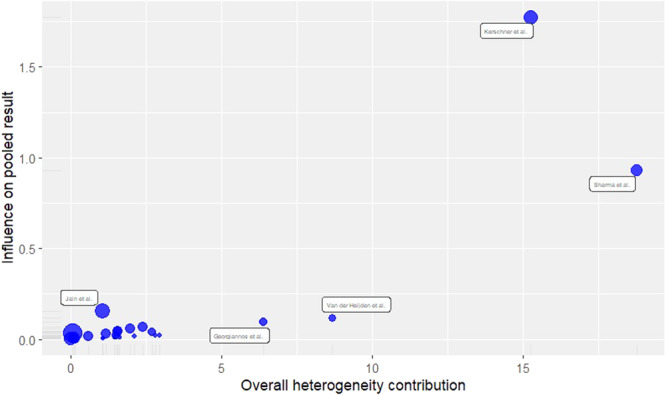
Influence analysis.

The influence analysis detected three outlier studies namely Kerschner et al. [19], Sharma and Cheng [41] and van der Heijden et al. [48]. After omitting the outlier studies the measures of heterogeneity saw a substantial decrease. *τ* ^2^ = 0.3024 (95% CI: 0–0.6273), *I* ^2^ = 25.9% (95% CI: 0%–54.7%), *Q* = 32.39, df = 24 and *p* value = 0.1175. The effects of these three studies are apparent in Figure 4.

#### Publication bias

**Figure 5:**
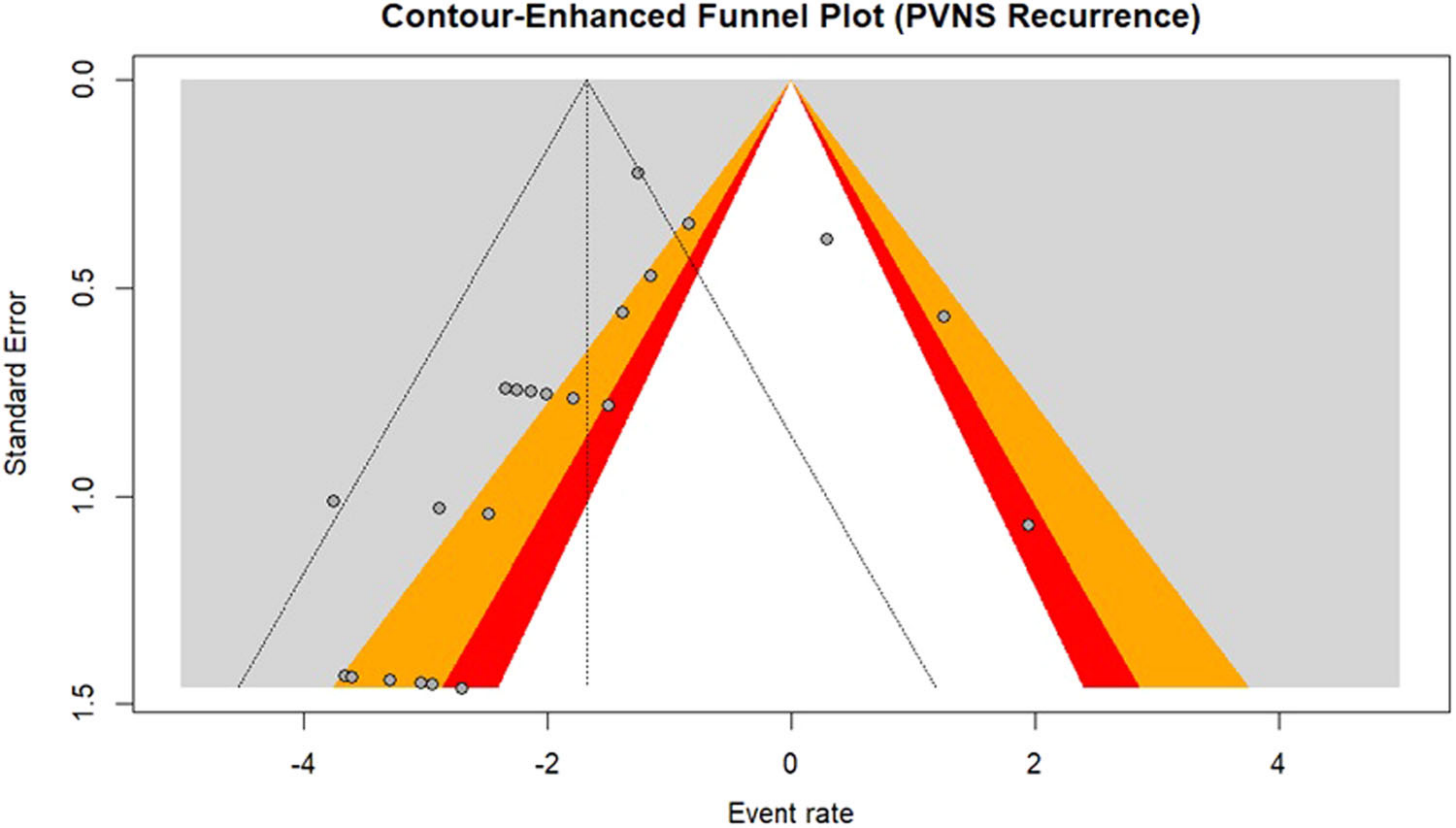
Contour‐enhanced funnel plot standard error for publication bias. Red: CI 90%, Orange: CI 95%, Grey: CI 99%. CI, confidence interval.

**Figure 6:**
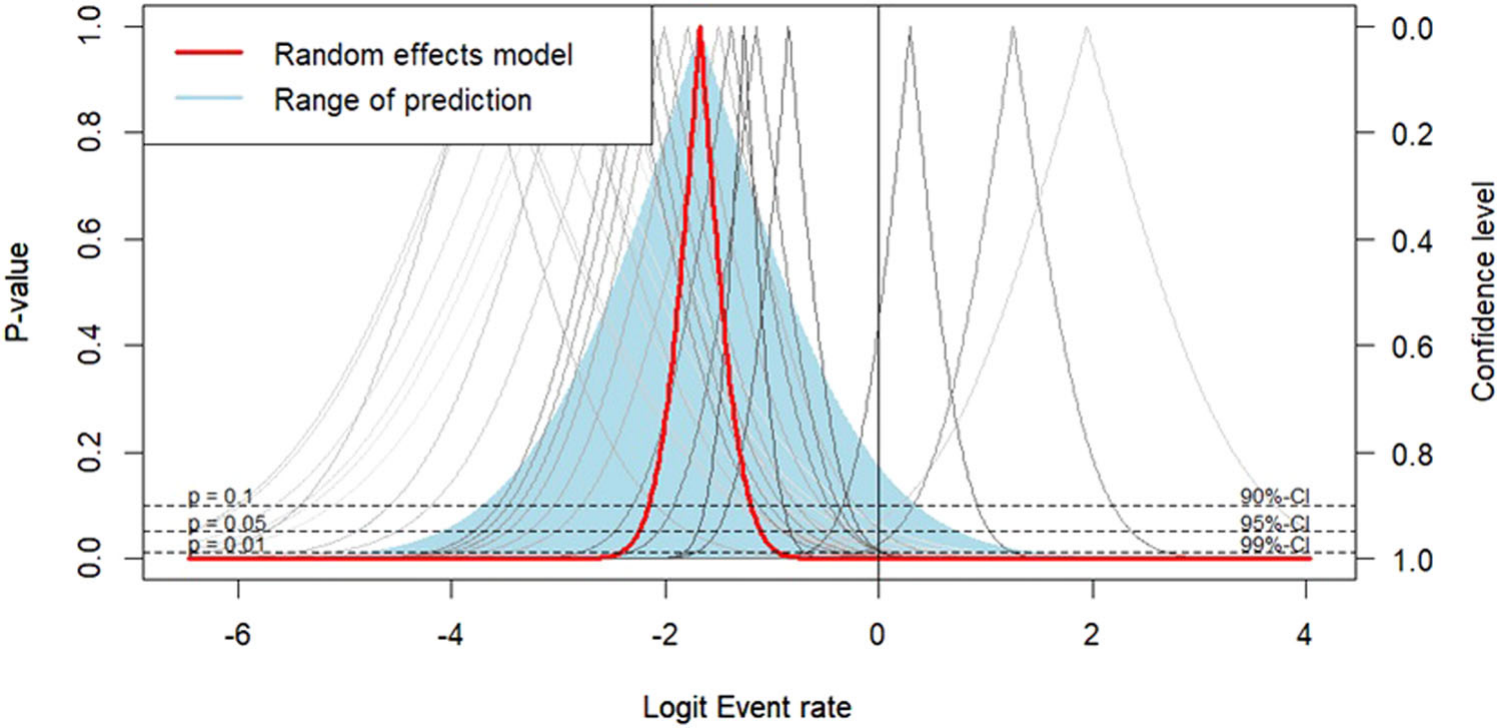
Drapery plot for publication bias.

Ultimately, a funnel plot was used to visually evaluate data symmetry (Figures 5 and 6), and Egger's test [11] was used to determine bias. The result of Egger's test revealed the existence of a publication bias (*p* value = 0.00318). The *p* value less than 0.05 implicates publication bias.

According to Egger's test, we found significant bias in the dispersion of data (Egger *p* < 0.05).

### Results of the meta‐analysis

#### Recurrence rates for PVNS

**Figure 7:**
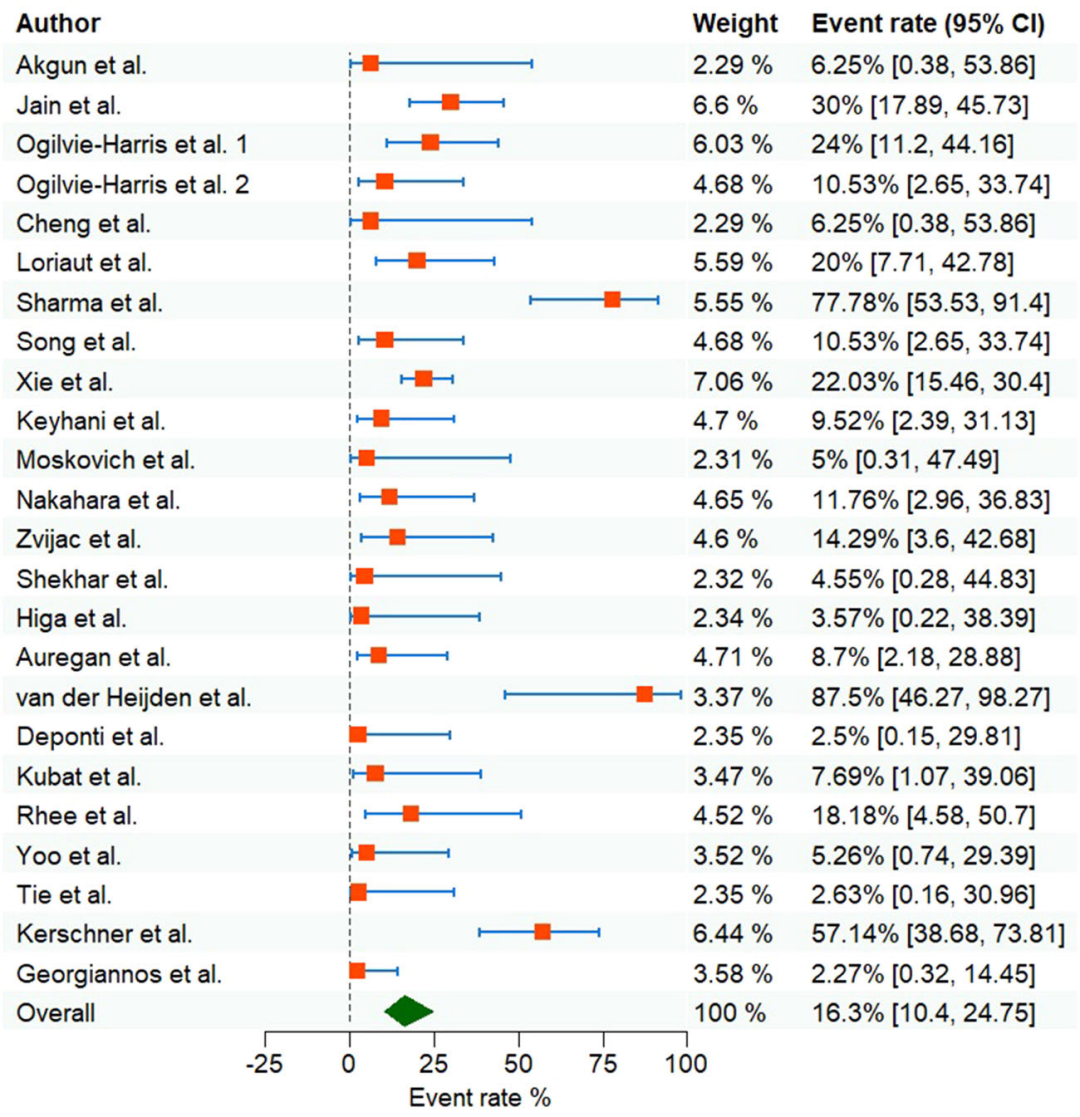
Forest plot of recurrence rate of PVNS. PVNS, pigmented villonodular synovitis.

A general meta‐analysis of all data indicated that the recurrence rate was 16.34% (95% CI: 10.4%–24.75%) (Figure 7). Therefore, based on this result, about 16% of arthroscopically operated knees were at risk of recurrence. Also, a sub‐group analysis and comparison of the recurrence rate in different subtypes of PVNS (Diffuse vs. Local) reported different values. A comparison of recurrence rates between these two subtypes showed that the diffuse subtype had a higher recurrence rate, 19.4% (95% CI: 10.01%–34.15%), in comparison with the localized subtype, 9.5% (95% CI: 4.47%–19.01%).

#### The course of recurrence rate by follow‐up period

**Figure 8:**
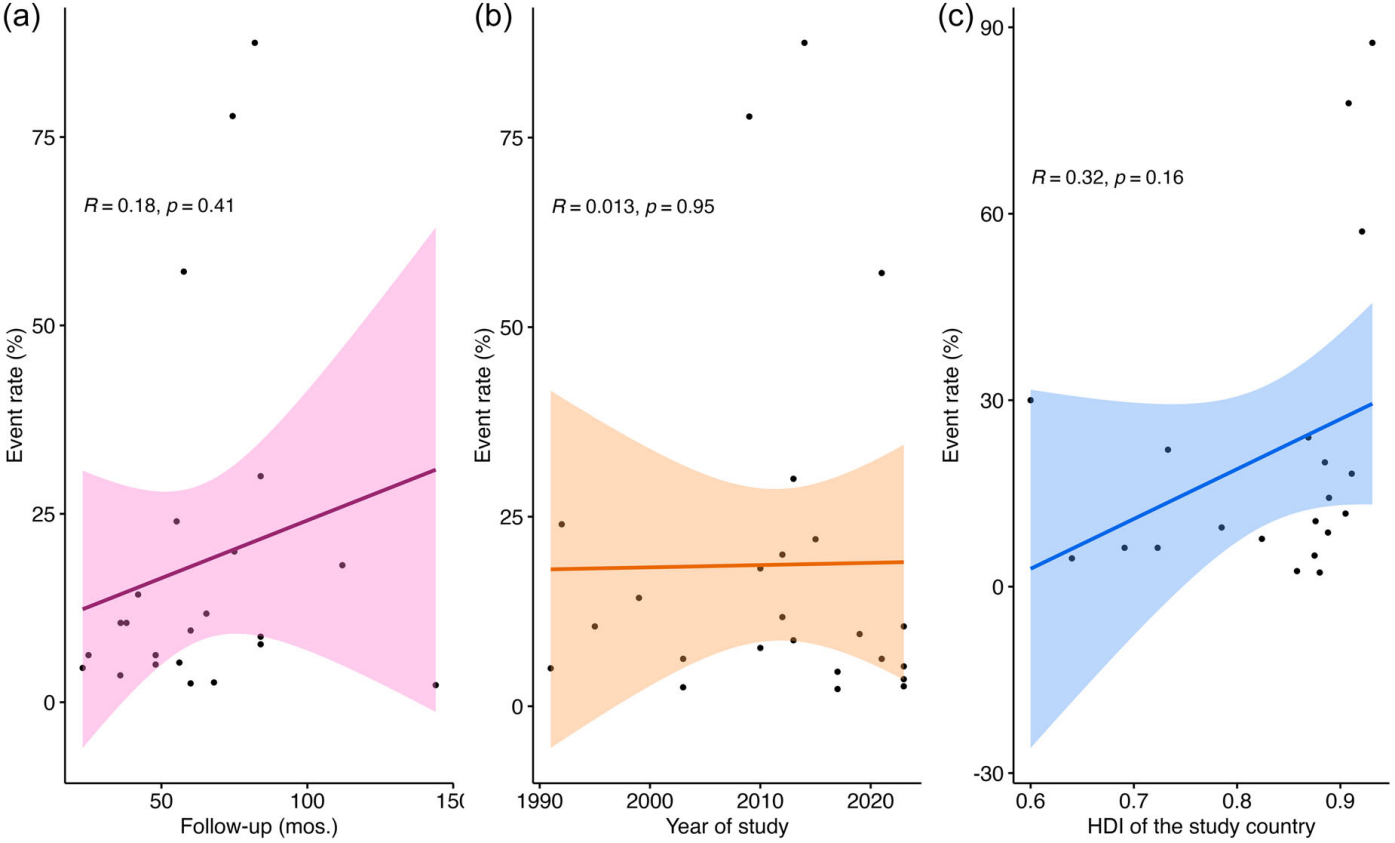
Meta‐regression of event rate (%) by (a) follow‐up period by months (%), (b) year of study, and (c) HDI of the study country. HDI, Human Development Index.

Meta‐regression analysis on the follow‐up period of the studies revealed a pronounced upward trend. The coefficient of the scatter plot was 0.18, *p* value = 0.41, (Figure 8a). Based on this result, the lower recurrence rate in the studies with shorter follow‐up periods can be attributed to the inadequacy of surveillance duration.

#### The course of recurrence rate in previous years

Meta‐regression analysis on the recurrence rate of previous studies revealed an insignificant slight growth in the recurrence rate of PVNS, which requires further evaluation of its causes in the future. The coefficient of the scatter plot was 0.013, and the *p* value = 0.95 (Figure 8b).

#### The course of recurrence rate by HDI

The HDI of the countries where the studies originated were extracted from the website of the United Nations Development Programme (https://hdr.undp.org/data-center/human-development-index). The meta‐regression revealed an upward trend in the recurrence rate. Coefficient: 5, *p* value >0.05. Alluding to the effects of higher health standards in developed countries on the recognition of the recurrence rate (Figure 8c).

#### The course of recurrence rate by mean age

**Figure 9:**
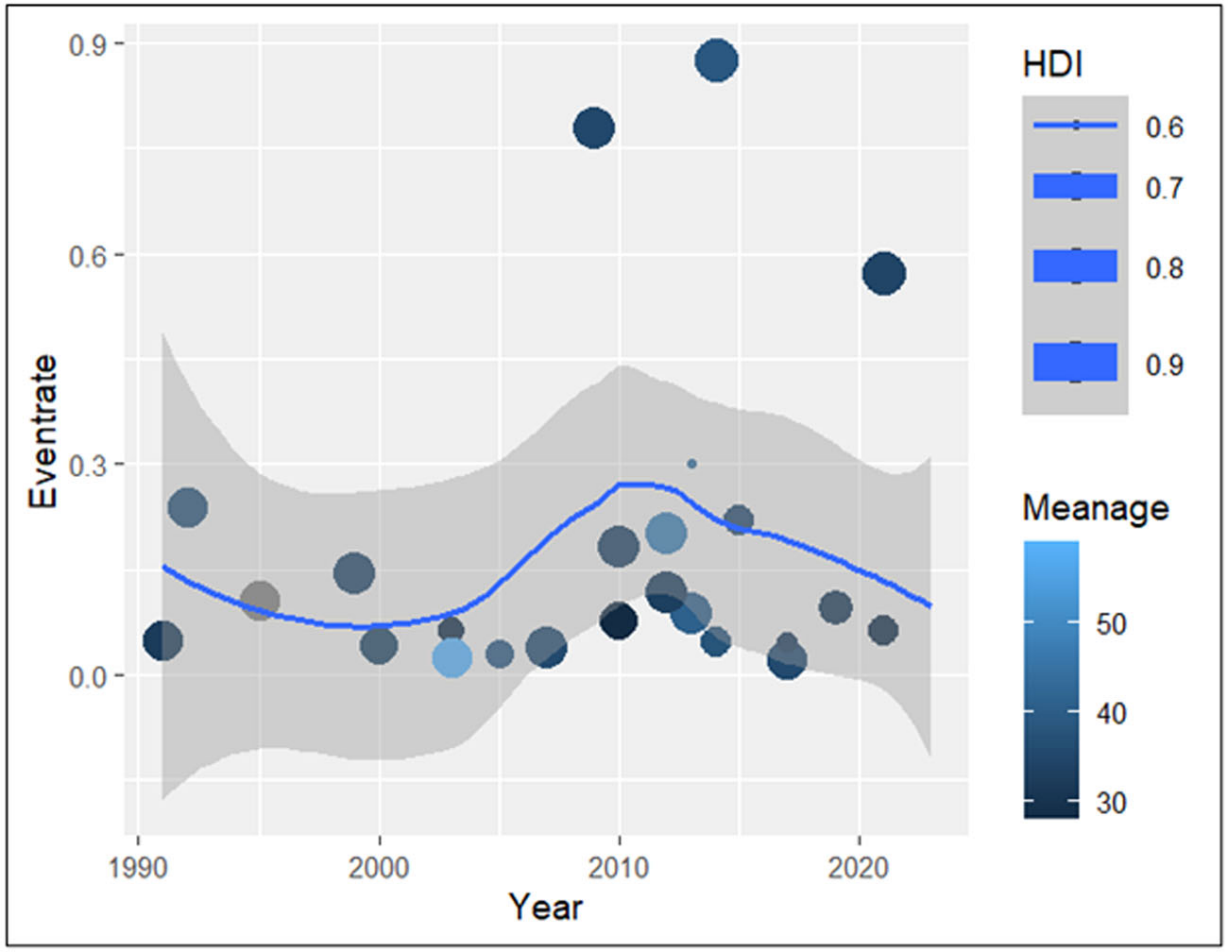
Scatter plot of standard error by point estimate for assessment of meta‐regression based on the HDI and mean age. HDI, Human Development Index.

Meta‐regression of the mean age did not reveal a direct cause‐effect interpretation. Coefficient: 0.0008, *p* value > 0.05 (95% CI: −0.11 to 0.11) (Figure 9).

## DISCUSSION

The most important finding of this study was that the diffuse subtype had a higher recurrence rate of 19.4% compared to the local subtype, which had a recurrence rate of 9.5%. In addition, despite the advantages of arthroscopic intervention, including reduced post‐operative complications and faster recovery times, the recurrence rate remains a concern, with about 16% of cases experiencing recurrence.

PVNS is a rare proliferative disease of the synovium with a treatment approach that lacks consensus and may carry a substantial risk of post‐operative complications and local recurrence. In recent years, there has been a growing trend towards the use of arthroscopic synovectomy in the treatment of PVNS of the knee. Based on our current knowledge, this systematic review and meta‐analysis represent the largest study pool investigating the effectiveness of the arthroscopic approach in reducing recurrence rates of PVNS of the knee. We incorporated cohort studies into our analysis to prospectively evaluate patients, thus minimizing the impact of recall and selection biases often encountered in retrospective case‐control studies. Additionally, we conducted a comprehensive search across five reputable databases to mitigate the risk of overlooking pertinent articles.

In our study, the recurrence rate for different subtypes of the disease was found to be 16.34%. According to the research conducted by Chandra et al., arthroscopic recurrence rates ranged from 11% to 17%. Their findings indicate that arthroscopic surgical treatment of DPVNS of the knee carries a 1.56 times greater risk of recurrence compared to open approaches. A study investigating arthroscopic versus open procedures revealed an annual recurrence rate (incidence) of 0.11 for arthroscopic procedures and 0.07 for open procedures. This equates to an absolute risk reduction of roughly 0.04 [4]. Some previous research has suggested that employing an open surgical approach leads to lower recurrence rates in comparison to using an arthroscopic approach for addressing DPVNS of the knee [37, 41, 48]. Aurégan et al. [3] analysis of local recurrence rates following open and arthroscopic surgery for both LPVNS and DPVNS revealed no statistically significant difference.

Notably, arthroscopic surgery for DPVNS was associated with a lower frequency of post‐operative complications. The precise cause of the higher recurrence rates in arthroscopic surgery remains uncertain. Prior studies have discussed potential reasons for the elevated recurrence rates in arthroscopic surgery, with some suggesting a link to inadequate removal of the neoplastic tissue [9, 41]. According to West et al., the overexpression of CSF‐1 in a small subset of cells is the leading cause of DPVNS. This finding supports the idea that incomplete resections, such as those performed with arthroscopic surgery, may result in the persistence of neoplastic driver cells, potentially raising the risk of recurrence [27, 52]. It is possible that arthroscopically shaving the synovium, rather than removing the neoplastic tissue as in open surgery, may lead to an increase in the level of CSF‐1 in the joint. This could occur due to a mechanical paracrine effect, where shaving neoplastic cells with high levels of intracellular CSF‐1 may result in cell destruction and the subsequent increased release of CSF‐1 into the joint. Mollon et al. observed a lower recurrence rate in a cohort treated with combined arthroscopic and open synovectomy compared to arthroscopic synovectomy alone. In their study, arthroscopic synovectomy alone was associated with the highest recurrence rate (37.8%). The increased use of arthroscopic surgery may have led to a trade‐off between the rate of recurrence and a decreased risk of post‐operative stiffness (2.1% vs. 10.5% in their series) [28]. Colman et al. [8] observed that among 48 patients with DPVNS of the knee who underwent treatment with arthroscopic (*n* = 26), open (*n* = 11) or combined approaches (*n* = 11), the recurrence rates were lower in the combined approach group compared to the arthroscopic or open approach groups (9% vs. 62% vs. 64%, respectively). Sharma and Cheng [41] conducted a study on eight patients with DPVNS of the knee who underwent a combined approach. Their findings did not yield conclusive results as they observed frequent recurrences irrespective of the surgical approach. It was noted that achieving sufficient gross surgical excision, better facilitated by an open approach, enhances local control using an open method [42]. The reports have discussed the advantages of an open approach, but the conclusions about recurrence rates were inconclusive due to the retrospective nature and small sample sizes of the studies. It is essential to understand the limitations of surgical resection for this disease in order to determine the most suitable candidates for this procedure. What is needed are large, long‐term prospective multicenter observational or comparative studies that focus on both recurrence rates and functionality. These studies would help reduce bias and provide a better understanding of the optimal management approach for PVNS.

In this study, the comparison of recurrence rates between the two subtypes showed that the diffuse subtype had a higher recurrence rate of 19.4% (95% CI: 10.01%–34.15%) compared to the local subtype, which had a recurrence rate of 9.5% (95% CI: 4.47%–19.01%). In a retrospective, multicenter study conducted by Mastboom et al. [25], it was found that there was no significant difference in the first local recurrence based on the surgical technique used in treating patients with DPVNS of the knee (*n* = 471, *p* = 0.11). However, other research groups have reported an increased risk associated with an arthroscopic approach. Although very low‐quality evidence suggests that the recurrence rate of LPVNS is unrelated to the surgical approach, for patients with DPVNS, low‐quality evidence indicates that the recurrence rate was reduced with both open synovectomy and combined open and arthroscopic synovectomy, as compared to arthroscopic surgery [28]. De Ponti et al. [9], reported that in the cases of LPVNS, arthroscopic local excision resulted in complete and persistent regression of the pathology. This underscores the importance of performing extended synovectomy in all cases of diffuse PVNS to ensure optimal outcomes.

The average follow‐up period in our study was 32.4 months (ranging from 25 to 144 months). Our results indicate that the lower recurrence rate in studies with shorter follow‐up periods may be due to insufficient surveillance duration. The length of follow‐up varies, and it may also identify recurrence despite the patient being asymptomatic.

This study is subject to certain limitations. The limitations of our review stem primarily from inherent biases in the case series. The studies included in our analysis had methodological limitations, such as small sample sizes in some trials, the use of different methods to screen for recurrence, and the inclusion of different populations. Due to the low incidence of PVNS and the extended period before recurrence occurs, it is challenging to conduct a prospective study with an adequate number of patients and sufficient follow‐up. Factors that could potentially influence recurrence rates, such as the expertise of the surgeon and the volume of the hospital, were difficult to control. Additionally, some reporting limitations made it challenging to separate data related to different treatments or LPVNS/DPVNS groups. It is important to note that our evaluation was limited to studies that involved an arthroscopic approach in the sample population.

The lack of consideration for the heterogeneity of patient cohorts in various studies can be attributed to the limited and moderate quality of the retrospective reviews available on this subject, as well as substantial bias across the studies. The study's findings were reported as annual incidence rates, suggesting that the overall rates of recurrence may be even higher when factoring in a longer follow‐up period.

### Conclusion

Our findings suggest that arthroscopic surgery for PVNS, particularly for the diffuse subtype, results in a higher recurrence rate compared to the localized subtype. However, the inherent challenges in achieving complete resection through arthroscopy, particularly in cases with extensive disease involvement, may contribute to the observed recurrence rates.

## AUTHOR CONTRIBUTIONS

Original idea: Sohrab Keyhani. Design, developing drafting and revision of the manuscript: Mehran Soleymanha. Contribution to the development of the protocol, data acquisition and analysis: Ali Nikibakhsh, Ervin Zadgari, Maryam Mousavi and Fardis Vosoughi. Final edition: Robert F. LaPrade. All authors read and approved the final manuscript.

## CONFLICT OF INTEREST STATEMENT

The authors declare no conflicts of interest.

## ETHICS STATEMENT

This study was approved by the Poursina Hospital Orthopedic Research Ethic Committee in Rasht, Iran.

## Supporting information

Supplementary information.

## Data Availability

The data sets used and analyzed during the current study are available from the corresponding author upon reasonable request.
