# Supplementary material for: Recurrence of arthroscopic treatment of pigmented villonodular synovitis of the knee: A systematic review and meta‐analysis
Source: J Exp Orthop. 2025 Feb 10;12(1):e70169. doi: 10.1002/jeo2.70169 (PMC11808254; doi:10.1002/jeo2.70169)
Supplement: Supplementary file 1 — Supplementary information. [file JEO2-12-e70169-s001.docx]

**Appendix 1. Search Strategies**

- **Web Of Science:**

**Strategy:**

**ALL=(pigmented villonodular synovitis) AND ALL=(Knee) AND ALL=(recurren*) AND ALL=(arthroscop*)**

**Date: 3.31.2024**

- **PubMed:**

**Strategy:**

**((((((("Giant Cell Tumor of Tendon Sheath"[Mesh] OR "Synovitis, Pigmented Villonodular"[Mesh]) AND (knee))**

**Number of results: 587b**

**Date: 3.31.2024**

- **Cochrane:**

**Strategy:**

**("pigmented villonodular synovitis" OR "tenosynovial giant cell tumor"):ti,ab,kw**

**Number of results: 42**

**Date: 3.26.2024**

- **Scopus:**

**Strategy:**

**TITLE-ABS-KEY ( "pigmented villonodular synovitis" ) AND ALL ( recurren* ) AND ALL ( knee ) AND ALL ( arthroscop* ) AND NOT TITLE ( hip ) AND NOT TITLE ( ankle ) AND NOT TITLE ( elbow ) AND NOT TITLE ( shoulder ) AND NOT TITLE ( wrist ) AND NOT TITLE ( mimic* )**

**Number of results: 376**

**Date: 3.28.2024**

- **Embase**

**Strategy:**

**'pigmented villonodular synovitis' AND 'knee' AND 'arthroscopic' AND 'recurrence*'**

**Number of results: 123**

**Date 3.29.2024**
